# A Predictive Model for Respiratory Failure and Determining the Risk Factors of Prolonged Mechanical Ventilation in Children with Guillain-Barre Syndrome

**Published:** 2020

**Authors:** Mohammad BARZEGAR, Vahideh TOOPCHIZADEH, Diena GOLALIZADEH, Ali PIRANI, Fatemeh JAHANJOO

**Affiliations:** 1 Pediatric Health Research Center, Tabriz University of Medical Sciences, Tabriz, Iran; 2 Physical Medicine and Rehabilitation Research Center, Tabriz University of Medical Sciences, Tabriz, Iran

**Keywords:** Guillain-Barre syndrome, predictors, respiratory failure, children, mechanical ventilation

## Abstract

**Objective:**

Determining the predictors of respiratory failure and duration of intubation in children with Guillain-Barre syndrome (GBS).

**Materials & Methods:**

Children diagnosed with GBS at Tabriz Children’s Hospital were studied. Factors associated with and influencing respiratory failure as well as the duration of intubation were determined using both univariate and multiple analyses.

**Results:**

Overall, 324 children were enrolled in the study, 54.0% of whom were males. Thirty-one (9.6%) patients underwent mechanical ventilation, the patients under 5 years old were more prone to the requirement of mechanical ventilation (11.3% vs. 6.9%). Cases hospitalized in winter were more likely to need ventilation compared to those hospitalized in spring (OR =7.00; 95% CI:1.51-32.53). Also, autonomic involvement (OR=8.88, 95% CI:4.03-19.58; p<0.001) and cranial nerves involvement (OR=9.88, 95% CI:3.68 - 26.52; p<0.001) emerged as risk factors for mechanical ventilation requirement. Overall, 16.1% of patients with axonal electrophysiologic pattern required mechanical ventilation compared to 7.4% of those with demyelinating type (OR:2.15, 95% CI: 1.01-4.69). In univariate analysis, the only variable that showed a correlation with the duration of intubation was axonal electrophysiologic pattern (p= 0.028).

**Conclusion:**

Approximately, 10% of the patients required mechanical ventilation. Season, cranial nerve involvement, autonomic dysfunction and electrophysiologic pattern were the most important variables in predicting respiratory failure and duration of mechanical ventilation.

## Introduction

Guillain–Barre syndrome (GBS) is considered the most common reason for acute neuromuscular paralysis in children. It is an immune-mediated and self-limiting disorder of neural tissues triggered by respiratory tract and gastrointestinal tract pathogens. The global annual incidence of GBS has been reported 0.66 to 1.8 cases per 100,000 children younger than 18 years old. Thus, GBS is known to be one of the most common causes of neuromuscular disease in this group, which presents with rapidly progressive weakness of the extremities and bulbar, autonomic dysfunction, paresthesia, pain and respiratory failure (1-3).

Respiratory insufficiency is the most serious and life-threatening complication associated with GBS in children, which occurs in 20-30% of pediatric patients requiring mechanical ventilation (MV). Many of the patients diagnosed with GBS admitted to the ICU and intubated are at risk for severe complications. The mortality rate in children with GBS is about 2-12%. Respiratory failure is the most common cause of death in these patients (4-8).

Unfortunately, 60% of patients who have been intubated and undergone MV were diagnosed with a major problem such as pneumonia, pulmonary embolism, sepsis, and gastrointestinal bleeding. In addition, the risk of aspiration pneumonia may increase in a patient with delayed intubation (6, 8).

Prediction of early MV in children with GBS, for the avoidance of aspiration, pneumonia, respiratory distress, septicemia and admission to ICU is necessary. Early MV may be helpful for better management and reduced complications and mortalities, and in turn, improved outcomes in such patients (6-8).

Also, mortality rate in patients with GBS whom have undergone MV varied from 8.3 to 20% (9), and the duration of MV in these patients ranges from a few days to even longer than several months. Because prolonged MV may lead to several complications such as damage to vocal cords and recurrent laryngeal nerves, if expected MV duration is more than two weeks (prolonged MV), early tracheostomy should be considered for the prevention of such damages. On the other hand, prediction of prolonged MV can curtail unnecessary early tracheostomy that causes infections, esophageal perforation, tracheal stenosis and scar (10).

Previous studies on adult patients showed various risk factors for MV and respiratory insufficiency in this group such as rapid progressive weakness, cranial nerves deficits and areflexia (6).

 To the best of our knowledge, there are limited studies with large sample sizes investigating the predictors of respiratory failure in childhood GBS. Thus, we aimed to perform a comprehensive evaluation of the predictors of respiratory failure. We sought to develop a model for the prediction of respiratory insufficiency and the risk factors for prolonged MV in children with GBS as a guide for decision-making on early intubation to reduce the associated major complications and improve outcomes in these patients.

## Materials & Methods

In this cross-sectional study, 324 children under 16 years old with GBS who had been admitted to Tabriz Children’s Hospital from 2003 to 2014 were enrolled. Informed consent was obtained from the parents. The Institutional Ethics Committee approved this research. GBS diagnosis was made based on Asbury and Cornblath criteria (11). 

Physical examinations and recording of clinical details were performed by a pediatric neurologist. The following data were recorded: age, gender, preillness, disability score, cranial involvement, autonomic dysfunction, CSF analysis parameters during hospital stay, need for mechanical ventilation, duration of intubation and electrophysiologic data. Autonomic dysfunction was evaluated by bedside clinical examination. Patients who developed arrhythmia, fluctuation in heart rate or blood pressure, pupillary abnormality, abnormal sweating, gastrointestinal and urinary dysfunction were considered to have autonomic dysfunction. 

Disability score was evaluated based on Hughes’s (12) criteria, based on which 0 represents normal and 6 is equal to death. 

Electrodiagnostic study was performed in all the patients during hospital stay by a pediatric electromyographer with the Medelec Synergy electromyography device. Sensory nerve action potential (SNAP) of the sural and median nerves and compound muscle action potential (CMAP) of the tibial, peroneal, median, and ulnar motor nerves were studied in all the patients. The amplitude of the negative phase for CMAPs and SNAPs, F-waves distal latency and nerve conduction velocity were obtained. The obtained data were compared with the normal values reported by Parano et al. (13).

Electrophysiologic parameters were classified according to Cornblath (14) definition as acute inflammatory demyelinating polyneuropathy (AIDP), acute motor sensory axonal neuropathy (AMSAN), acute motor axonal neuropathy (AMAN), Miller-Fisher syndrome and unclassified subgroups. If the following changes were observed in at least two nerves, the patients were classified as having AIDP: prolongation of distal latency >125% of the normal upper limit, reduced nerve conduction velocity <80% of the normal lower limit, f-wave prolongation >120% of the normal upper limit, conduction block and temporal dispersion >15% increase in negative phase duration. 

Patients with reduced compound muscle action potential (CMAP) amplitudes without evidence of demyelination based on the above-mentioned criteria were considered as AMAN. When there were reduced CMAP and sensory nerve action potential (SNAP) amplitudes, the patient was classified as AMSAN. Those with low-amplitude SNAPs and the clinical triad of ataxia, ophthalmoplegia, and areflexia were classified as the Miller-Fisher variant (14).

The decision to start mechanical ventilation was based on the presence of: respiratory distress, PaCo_2_ >60 torr, Po_2_ <60 torr, pH <7.25, inefficient cough and severe bulbar dysfunction (8, 15, 16).

Based on the aim of this study, the patients were compared according to the need for mechanical ventilation and duration of intubation.

**Statistical Analysis**

For analysis, SPSS version 16.0 was used. In the descriptive statistics section, mean and standard deviation were used for quantitative variables and frequency and percentage was used for qualitative variables. The study factors were compared between those who received mechanical ventilation and those who did not by using Chi-square test or, if necessary, using Fisher’s exact test. Then, the variables that were significant at the level of 0.2 were considered as potential factors in predicting the need for ventilation and were entered into a multiple logistic regression model. At this stage, odds ratio and confidence interval for the odds ratio of all the variables were calculated and reported.

To explore difference in mechanical ventilation duration in different categories of independent variables, Mann-Whitney U test or Kruskal Wallis test was used. The negative binomial regression test was performed to predict the duration of ventilation. 

## Results

In this study, 324 patients admitted to Tabriz Children Hospital with GBS diagnosis were enrolled, of whom 175 (54.0%) were male. Their mean age was 5.13 ± 3.66 years (6 months to 16 years old).

 The results of baseline characteristics showed that GBS was more prevalent in children under the age of 5 years and in summer. Also, upper respiratory tract infection was the most commonly reported illness that occurred before the onset of paralysis. 

Thirty-one (9.6%) patients underwent mechanical ventilation, mean duration of intubation was 20.75 ± 21.25 days and mortality rate was 1.5%. Although age and mechanical ventilation association did not reach statistical significance, the patients under 5 years old were more prone to need for mechanical ventilation (11.3% vs. 6.9%). The predictors of mechanical ventilation requirement were seasonal prevalence (16.5% in winter vs. 2.8% in spring; OR: 7.00, 1.51 - 32.53, p=0.04). In addition, children with autonomic nervous system involvement were significantly (8.88 times) were more likely to require mechanical ventilation than the ones without autonomic nerve involvement. One hundred twenty-seven patients had cranial nerves involvement during hospital stay (cranial nerve III:3.8%, VI:6.5%, VII:20.8%, IX:34.4%, X:32.1%, and XI:2.3%). Those with cranial nerve involvement had significantly greater need for mechanical ventilation in comparison to those without cranial nerve palsy (20.4% vs. 2.5 %, respectively; OR: 9.88; 95% CI: 3.68-26.52; p<0.001).

 Analysis of CSF was performed in the first week after admission, protein levels were included in the analysis. Overall, 75.2% of the patients had protein levels equal to or greater than 40 mg/dl, all the normal analyses were performed in the first week of the disease.

Electrophysiologic evaluation was performed during hospital stay between 3 and 21 days after onset of weakness (5.85 days on average). Electrophysiologic pattern includes: AIDP 45.6%, AMAN 36.1%, AMSAN 2.16%, Miller Fisher 1.5%, pharygo cervico brachial 0.3%, unclassified 3.2% and normal 11.1%.

Overall, 16.1% of the patients with axonal type nerve injury (AMAN) required mechanical ventilation compared to 7.4% of those with demyelinating type (AIDP), OR:2.15, (1.01 - 4.69). The need for mechanical ventilation was also related to CMAP amplitude (6.8 if low vs. 32.1% if absent; OR: 6.47, 2.4-17.36, p<0.001; Table 1).

The most common complication was pneumonia which was found in 17 (5.2%) patients. GI bleeding was seen in 8 (2.5 %) patients. Other complications were found in 6 patients (collapse – 5 and atelectasis - 1), and pneumonia and collapse were reported in eight (2.5%) patients simultaneously. 

**Anticipating Mechanical Ventilation Requirement**

To identify variables predicting the need for mechanical ventilation, variables that were significant in univariate analysis at the level of 0.2 (age, p =0.19; gender, p = 0.92; seas,on p = 0.04; treatment, p = 0.001; sensory, p = 0.20; autonomic involvement, p < 0.001; cranial involvement, p = 0.001; electrophysiological pattern, p = 0.016 and CMAP) were selected and entered into the multiple logistic regression model.

The complications and treatment variables were not modeled due to overlapping. Also, CMAP and EMGNCV variables were excluded from the model due to affecting the accuracy of the prediction model and leading to biased estimates.

The results of multiple logistic regression showed that among variables that entered the model in the first step (i.e., age, gender, season, sensory, autonomic and cranial involvement), season with p = 0.04, autonomic involvement with p < 0.001 and cranial involvement with p <0.001 were entered into the final model. The final model showed that the cases hospitalized in winter are seven times more likely to need ventilation compared to the cases hospitalized in spring (OR =7.00; 95% CI:1.51-32.53). The odds ratio for vanishing in the event of autonomic involvement was approximately nine times the odds ratio of hospitalization in the absence of autonomic involvement. The need for mechanical ventilation was approximately 10 times higher in cases with cranial involvement (OR =9.88, 95% CI:3.68 - 26.52).

Regarding the above results, the final regression model for predicting mechanical ventilation requirement can be presented as follows:

Ln p1-p=-5.64+2.25 winter+1.288 fall+1.128 summer+2.278 autum+2.286 cranial involvment

So that:

pMV=e-5.64+2.25 winter+1.288 fall+1.128 summer+2.278 autonum+2.286 cranial involvment 1+e-5.64+2.25 winter+1.288 fall+1.128 summer+2.278 autonum+2.286 cranial involvment 

Where:

 P: probability

 Ln: log base 10

 e: exponential

For example:

The probability of need for ventilation in a child referred in spring with no autonomic or cranial involvement is 0.004. The probability of need for ventilation in a child referred in spring with autonomic and cranial involvement is 0.254

**Multiple logistic regression model goodness of fit**

 The results presented in Table 2 show that using the three independent variables included in this model (i.e., season, autonomic and cranial involvement), we can explain the variation of the dependent variable (need for ventilation) with a confidence of 90.3%. The values of the Cox and Snell and Nagelkerke determination coefficients are equal to 0.180 and 0.384, indicating that between 18 to 38%bof the variability of the dependent variable can be determined by this set of independent variables. The Omnibus index at the confidence level of 0.99 and the Hamster and Lemeshow index at the confidence level of 0.95 represent the best-fitting model with explanatory factors (Table 2).

**Figure 1:**
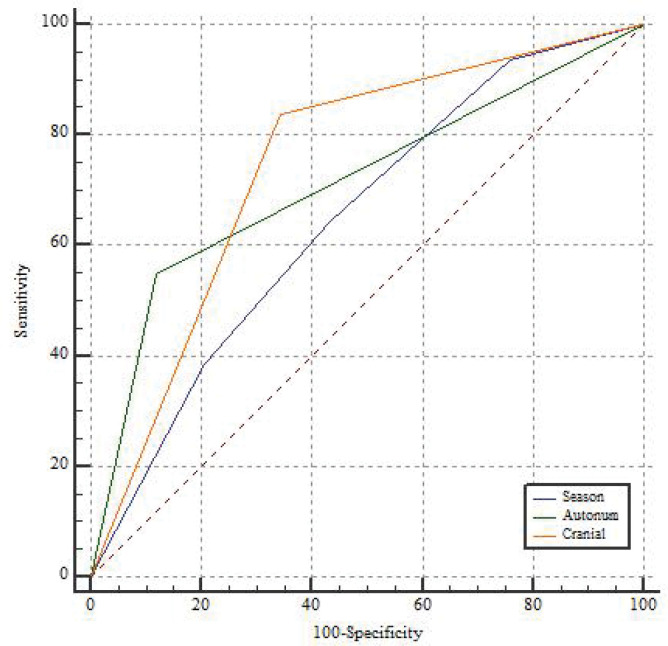
Receiver operating characteristic curve based on logistic regression including season, autonomic involvement and cranial involvement

Also, for estimating the accuracy of the fitted model, the area under the ROC curve was used. The area under the ROC curve for season, autonomic involvement, and cranial involvement variables is 0.647, 0.714, and 0.747, respectively, which indicates the diagnostic power of the three variables in the prediction of the need for ventilation. The sensitivity of season is 64.52%, autonomic involvement is 54.48% and cranial involvement is 83.8%, which indicates high accuracy. Also, the specificity of 56.36 %, 87.97 % and 65.52 % for the season, autonomic involvement and cranial involvement variables, respectively, indicates high accuracy of Figure 1. 

**Duration of Mechanical Ventilation**

The results shown in Table 3 reveal that in univariate analysis the only variable correlated with the duration of intubation is axonal electrophysiologic pattern (AMAN; p= 0.028).

To predict the duration of ventilation, variables that were significant at the level of 0.2 in univariate analysis were season (p = 0.075), treatment (p = 0.172), autonomic involvement (p = 0.173) and electrophysiological pattern (p = 0.023; Table 3), which were selected and entered into the negative binomial regression model. Only electrophysiological pattern was significant.

**Table 1:** Main demographic and clinical characteristics to identify patients who did and did not require mechanical ventilation

Variables		MV+	P-value	Odds ratio (95%CI)
Age (Year)	<= 5 Y 202	23 (11.3%)	Chi square 0.19	1.72 (0.74 - 3.99)
	> =6 Y 122	8 (6.5%)		Reference group
Gender	Male 175	17 (9.7%)	Chi square 0.92	1.04 (0.49 - 2.19)
	Female 149	14 (9.4%)		Reference group
Season	Spring 72	2 (2.8%)	Chi square 0.04*	Reference group
	Summer 104	9 (8.7%)		3.35 (0.70 - 15.99)
	Fall 75	8 (10.7%)		4.17 (0.86 - 20.40)
	Winter 73	12 (16.5)		7.00 (1.51 - 32.53)*
preillness	AURI 134	16(12%)	Chi square 0.40	Reference group
	GE 50	4 (8.0%)		0.63 (0.20 - 1.99)
	Other 11	2 (18.2%)		1.61 ( 0.32 - 8.13)
	None 129	9 (7.0%)		0.55 (0.23 - 1.30)
Sensory/pain	No 175	20 (11.4%)	Chi square 0.20	1.65 (0.76 - 3.56)
	Yes 149	11 (7.4%)		Reference group
Autonomic Involvement	No 271	14 (5.2%)	Chi square <0.001*	Reference group
	Yes 53	17 (32.0%)		8.88 (4.03 - 19.58)*
Cranial Involvement	No 197	5 (2.5%)	Chi square <0.001*	Reference group
	Yes 127	26 (20.4%)		9.88 (3.68 - 26.52)*
Electrophysiological pattern	Normal 36	0 (0.0%)	Exact test = 0.016*	0.29 (0.009 - 2.68)
	unclassified 10	0 (0.0%)		0.98 (0.03 - 9.75)
	AIDP 148	11 (7.4%)		Reference group
	Axonal 124	20 (16.1%)		2.15 (1.01 - 4.69)*
CMAP Amplitude	>50%LLN 119	0 (0.0%)	Exact test <0.001*	0.39 (0.01 - 3.62)
	<50%LLN 177	12 (6.8%)		Reference group
	Absent 28	9 (32.1%)		(2.41 - 17.36)*

MV+: patients underwent mechanical ventilation

**Table 2:** Multiple logistic regression model goodness of fit indicators with the presence of predictive variables

Hosmer and Lemeshow Test		Omnibus Tests		Model Summary		Total percentage of prediction accuracy
P-value	Chi-square	P-value	Chi-square	Nagelkerke R Square	Cox & Snell R Square	90.3
0.344	6.754*	<0.001	63.81**	0.384	0.180	

*confidence level 95%, ** 99% confidence level

**Table 3:** Main demographic and clinical characteristics affecting the duration of mechanical ventilation

Variables		Duration of MV (days) Mean ± SD	P-value
Age (Year)	<= 5 Y	22.24 ± 4.89	Mann-Whitney U test 0.796
	> =6 Y	16.29 ± 6.82	
Gender	Male	17.80 ± 5.56	Mann-Whitney U test 0.235
	Female	24.15 ± 5.85	
Season	Spring	25.50 ± 12.50	Kruskal Wallis test 0.075
	Summer	7.75 ± 3.32	
	Fall	34.43 ± 11.76	
	Winter	20.64 ± 5.00	
preillness	AURI	20.47 ± 5.42	Kruskal Wallis test 0.225
	GE	7.50 ± 3.20	
	Other	34.00 ± 4.00	
	None	25.14 ± 10.56	
Sensory	No	18.11 ± 4.57	Mann-Whitney U test 0.408
	Yes	25.50 ± 7.79	
Autonomic Involvement	No	20.50 ± 5.67	Mann-Whitney U test 0.667
	Yes	21.00 ± 5.90	
Cranial Involvement	No	8.40 ± 2.87	Mann-Whitney U test 0.173
	Yes	23.43 ± 4.68	
Electrophysiological pattern	Normal	-	Kruskal Wallis test 0.023*
	Nonspecific	-	
	AIDP	8.22 ± 2.51	
	Axonal	26.68 ± 5.31	
CMAP Amplitude	Normal	-	Kruskal Wallis test 0.331
	Low	16.44 ± 6.00	
	Absent	29.00 ± 9.57	

* Level of significance is considered to be <0.05.

## Discussion

This study reports the predictors of respiratory failure caused by childhood GBS in Iran. The main strengths of this research are the large number of patients included and pediatric patient study. In this study, data of 324 pediatric GBS patients were analyzed to recognize the main risk factors for the need for MV and duration of MV using an accurate model.

Based on the results of this study, GBS is more prevalent in children under the age of 5 years, and approximately 10% of such patients undergo mechanical ventilation. 

Season, autonomic dysfunction and cranial nerve involvement were the important risk factors for the prediction of need for MV, based on these factors an accurate model with a confidence of 90.3% was made to estimate the rate of requirement of MV in children because it has been proved that early MV could reduce complications (4-6).

There are two important issues in the field of GBS management, that is, short-term poor prognosis (acute respiratory failure and death) and long-term poor prognosis (delay in independent walking ability). Differences in mechanical ventilation rate could be due to different study populationss, pathogens, supportive care, and the decision to start artificial ventilation (18).

It seems that weakness in facial, oropharyngeal, retropharyngeal, and respiratory muscles in addition to secondary complications such as pneumonia or alveolar collapse are responsible for respiratory failure in GBS (19,20). GBS may lead to bulbar paralysis, ophthalmoplegia, and lingual muscles weakness. Facial and shoulder muscle paralysis in accordance with dysphagia are the risk factors for respiratory failure (21). In the present study, the most involved (43.3%) cranial nerves were 9 and 10.

Younger age of the disease was a risk factor for respiratory failure (18, 21), in this study patients under 5 years old were more prone to require mechanical ventilation. 

We found no significant relationship between preceding infection and respiratory failure. However, some studies suggested that if the duration between preceding infection and weakness onset was shorter than 8 days, respiratory failure is probable (21)

Autonomic nervous system involvement is common in GBS. There are different reports of frequency of dysautonomia in GBS. Nonetheless, approximately 16.3% of the patients in this study had dysautonomia, which is a major cause of death in GBS patients and is manifested by sympathetic and parasympathetic disturbances (17, 18, 22). Based on our knowledge, there are few studies with large sample sizes on GBS among the pediatric population in the literature. In a study of 155 GBS adult patients in 2001, 30% experienced respiratory failure. Similar to the results of the current study, patients with rapid progressive course, bulbar palsy, bilateral facial nerve, and autonomic nervous system involvement were more susceptible to respiratory failure. Additionally, vital capacity below 20 ml/kg and maximal respiratory pressure below 30 cm (H_2_O) were associated with respiratory failure (23-25). Pulmonary function tests were not performed in our study due to the young age of the patients.

A study in 2003 evaluated mechanical ventilation risk factors among GBS patients. Individuals with inabilities to cough, stand up, and raise their elbows and heads from the bed and over the seven-day admission duration were associated with more frequent respiratory failures. Authors in this study believed that if four of these six mentioned disabilities were present, ICU admission is mandatory. In their study, younger age was associated with higher risk for respiratory failure and swallowing disorder (26). Since weakness progression in GBS has an ascending pattern starting from lumbar spinal nerves toward cervical and cranial nerves, it is reasonable to conclude that cervical region involvement including C4 root would lead to diaphragmatic muscle weakness and subsequent respiratory failure. We found higher rates of respiratory failure in GBS patients with the 9^th^ and 10^th^ cranial nerve involvement (p<0.00). This finding is in line with the results of previous studies.

Electrophysiologic pattern and absent or unobtainable CMAP on nerve conduction study which could be due to axonal degeneration or conduction block were among the predictors of poor outcome in this and other studies (24, 27, 28). The incidence rates of AIDP and AMAN forms of GBS vary in different regions, which may be due to genetic background and inciting pathogens. There was a significant correlation between AMAN pattern, CMAP amplitude, and outcome in this study. 

Electrophysiological pattern (axonal) was a significant risk factor for prolonged MV in our study. Therefore, on admission, this prognostic factor in children with GBS should be assessed to reduce the side effects of late or early MV and avoid unnecessary intubation. Although GBS is a self-limiting disease, complications of MV could be serious and life-threatening (4-6).

In a review and meta-analysis study of GBS patients from all age groups, it was demonstrated that short interval between onset of weakness and admission to hospital (less than seven days), bulbar and neck weakness and severe muscular weakness were more related to the requirement of MV and intubation, and facial weakness and autonomic disturbance were observed more significantly in patients with respiratory failure (29). Results of this meta-analysis were in line with our findings. Also, season and axonal pattern have not been reported in any study, but these variables in our study showed a significant relation with the need for intubation in children.

 The study performed among 40 children with GBS which predicted the risk factors for respiratory failure demonstrated a significant association between the need for MV and Hughes score at nadir, autonomic dysfunction, respiratory distress, hypotension, consciousness disturbance and the AMSAN form of GBS (4). In our study, the AMAN form of GBS had a greater association with the duration of MV, while other risk factors did not have a significant relationship with MV in children with GBS.

In a study, local demyelination in electrophysiological testing was considered a prognostic factor for the need for MV in GBS (30), but in our study, axonal electrophysiological pattern (SD=26.68 ± 5.31) and CMAP amplitude decrement (SD= 29.00± 9.57) were more related with indication and duration of MV in children with GBS.

According to an investigation on adult patients with GBS, some variables such as facial nerve palsy, short interval between onset of symptoms and admission, Vagus and Glossopharyngeal nerves involvement, bulbar weakness and Medical Research Council Sum score were important predictive factors for the need and duration of MV (6, 7).

In this study, cranial nerve involvement (OR=11.09:95% CI: 3.02-40.75) in children was a risk factor for MV requirement. Furthermore, axonal-type GBS had a greater relation with the duration of MV in pediatric patients. However, Durand et al. demonstrated demyelinating form of GBS, proximal to distal CMAP amplitude decrement ratio of peroneal nerve and vital capacity were more important predictive factors for the need for MV and intubation in adult patients (30).

In another research, bulbar dysfunction, autonomic disorder and upper limbs paralysis were significant predictors of respiratory failure in adult patients with GBS (5), but these variables were not established predictive factors in our observation of children and these factors were not significant among mechanically ventilated or non-ventilated patients.

In a retrospective study performed among 750 adult and pediatric GBS patients to compare these groups of patients, researchers found that autonomic dysfunction was an independent important risk factor for predicting MV in children. Of course, clinical findings differed in the two groups(31). The results of this study were similar to our findings.

In another study on 369 patients, the average duration of MV was 21 days and autonomic dysfunction and facial and bulbar weakness, high transaminase level and severe weakness were related to higher frequency of MV. In that study, 26.6% of ventilated patients (similar to non-ventilated patients) had poor outcomes, but compared to non-ventilated patients, ventilated patients had more complications and longer hospitalization periods (32).

In a cross-sectional study by Sundar et al. who compared clinical findings of ventilated and non-ventilated patients, they understood that quick progression to highest disability, bulbar and autonomic dysfunction and the axonal form of GBS predicted the progress of respiratory distress in GBS (25). These data in adult patients are consistent with our findings in children.

Analysis of 733 adult patients with GBS revealed that 313 (43%) patients underwent endotracheal MV. More than 85% of the patients had at least four of the following risk factors: interval between the onset of weakness and admission< 7days, inability to cough, stand, lift the elbow or head, liver enzyme increase and vital capacity <60%, required MV. Thus, patients with even one of these factors should be supervised in ICU (26). Although the results of this survey were interesting, but different predictive factors were assessed which is difficult to examine in children.

**In Conclusion,** This study has some limitations; we could not evaluate some variables including rapidity of disease onset, admission time, early treatment and detailed muscle strength in the upper and lower limbs, because they were not clearly documented in patients’ charts. In addition, the effects of treatment (immunoglobulin or plasma exchange) were not assessed in this cross-sectional study, and we could not statistically analyze AMSAN subtype separately due to the limited number of these cases. 

**In Conclusion**, Based on the findings of this study conducted among 324 children with GBS, season, cranial nerve involvement, autonomic dysfunction and electrophysiological pattern are the most important variables for the prediction of respiratory failure, duration and need for MV and intubation in children with GBS. Therefore, these variables should be evaluated in all patients at the time of admission and calculate the possibility of MV and duration of MV using models based on these main variables.
